# Clinical ethics consultation documentation in the era of open notes

**DOI:** 10.1186/s12910-023-00904-1

**Published:** 2023-05-03

**Authors:** Chad Childers, Jonathan Marron, Elaine C. Meyer, Gregory A. Abel

**Affiliations:** 1 Marian University College of Osteopathic Medicine, 3200 Cold Spring Rd, 46222 Indianapolis, IN USA; 2 Center for Bioethics, Harvard Medical School, 641 Huntington Ave, 02115 Boston, MA USA; 3 Division of Population Sciences, Dana-Farber Cancer Institute, 450 Brookline Ave, 02215 Boston, MA USA; 4 Dana-Farber/Boston Children’s Cancer and Blood Disorders Center, 450 Brookline Avenue, 02215 Boston, MA USA

**Keywords:** Open Notes, Ethics Consultation, Narrative Medicine, Clinical Ethics, Ethics Documentation

## Abstract

**Background:**

In 2021, federal rules from the 21st Century Cures Act mandated most clinical notes be made available in real-time, online, and free of charge to patients, a practice often referred to as “open notes.” This legislation was passed to support medical information transparency and reinforce trust in the clinician-patient relationship; however, it created additional complexities in that relationship and raises questions of what should be included in notes intended to be read by both clinicians and patients.

**Main Body:**

Even prior to open notes, how an ethics consultant should document a clinical ethics consultation was widely debated as there can be competing interests, differing moral values, and disagreement about pertinent medical information in any given encounter. Patients can now access documentation of these discussions through online portals which broach sensitive topics related to end-of-life care, autonomy, religious/cultural conflict, veracity, confidentiality, and many others. Clinical ethics consultation notes must be ethically robust, accurate, and helpful for healthcare workers and ethics committee members, but now also sensitive to the needs of patients and family members who can read them in real-time.

**Conclusion:**

We explore implications of open notes for ethics consultation, review clinical ethics consultation documentation styles, and offer recommendations for documentation in this new era.

## Background

In 2016, the 21st Century Cures Act [1] was passed which required that by April 5, 2021, eight different types of clinical notes be made available in real-time, online, and free of charge to patients. This practice can be referred to as “open notes;” it allows patients to rapidly access consultation notes, history and physical notes, discharge summaries, imaging narratives, laboratory report narratives, pathology report narratives, procedure notes, and progress notes. The legislation was passed with the hope of strengthening trust in the clinician-patient relationship and providing patients with more voice and responsibility over their electronic health records, and in their overall care. Though, it added a new component to the clinician-patient relationship and raises questions of what should, or should not, be included in notes intended to be read by many parties. Moreover, open notes have the potential to further complicate complex clinical cases that involve many stakeholders, a problem which commonly exists in clinical ethics consultations (CEC) [2].

While CECs can serve many purposes, they are often used to make ethical issues clear, encourage discussion, and/or provide guidance that supports stakeholders’ values and beliefs [3, 4]. Documentation of these encounters is critical, both to provide a record of what was discussed and to help educate the clinical teams involved. Even prior to open notes, there was debate about how CEC documentation should occur [5]. With the advent of open notes there are additional readers (e.g., patients, family) who need to be considered, adding greater complexity to the documentation issue.

In any given CEC encounter, there can be competing interests, differing moral values, disagreement about pertinent medical information, and countless other conflicts and/or disputes. Patients can now access documentation of these discussions through online portals which broach sensitive topics related to end-of-life care, autonomy, religious/cultural conflict, veracity, confidentiality, and others [6]. The breadth of ethically complex issues that CECs address can be exacerbated by the procedural and technical challenges that arise from the creation of the note itself, the necessity of accessing notes through online patient portals, privacy laws, and institutional standards and regulations. These practical concerns influence and inform the ethical complications inherent in CECs by hindering—or potentially even enhancing—communication amongst the healthcare team, patients, and family members.

Open CEC notes must be designed not only to be ethically robust, practical to record and access, accurate, and helpful for healthcare workers and ethicists, but also attentive to the needs of patients and their family members. Below, we explore benefits and implications of open notes for ethics consultation, review CEC documentation styles and current standards, and explore recommendations for CEC documentation in the era of open notes.

## Open notes

In 2010, Delbanco and colleagues [2] presented some of the first data characterizing the benefits and disadvantages of sharing clinical notes with patients. The project enrolled over 100 primary care physicians covering 25,000 patient participants. Patients were asked to access their visit notes through email and read them in a secure online portal. The project was designed to determine whether the open notes process was beneficial for patients and physicians alike, and if such a practice could serve as standard procedure in the American healthcare setting. The initial results from the project were positive enough to encourage future studies as well as eventual legislative action. Potential benefits included patient education, improved communication, and empowerment; however, the investigators also uncovered downsides such as additional time spent on notes by physicians, disagreement about medical information with patients, and even occasional feelings of “embarrassment” by treating physicians regarding their writing ability. These results were supported by a follow-up study which showed 99% of patient participants (n = 5219) wanted open notes to continue and none of the participating physicians elected to stop the practice [7].

Analysis from a 2019 study of open notes covering 23,000 potential patient participants from three different regions of the U.S. found that patients from historically marginalized populations benefited most from open notes [8]. Patients who were older, from minority groups, and not native English speakers reported the most value. Very few patients reported being confused about their notes (3.3%), and other benefits included help in remembering plans of care and feeling better supported to prepare for future visits. The investigators concluded that those vulnerable patients most needing thorough communication and the ability to have their voice recorded accurately were reaping advantages of this new practice. These findings suggest that medically complex information is not always immediately heard or understood by patients, and open notes may allow patients to revisit their medical information to process details that were missed.

Although, the investigators also found challenges as open notes were implemented across healthcare systems. Nearly half of participating patients did not enroll in the patient portal, and many were not aware that their notes were available. These findings highlight the need for healthcare providers to ensure their patients are informed and able to access their notes. The results should also be interpreted with caution because patient-participants self-selected and therefore were potentially more health literate. Indeed, a study of Veterans Administration open notes users (n = 37,103) revealed that 27.2% of participants found information “difficult to understand” [9]. Health literacy and barriers to access can present challenges for patients who may be less technologically savvy or have limited access to computers and the internet. This poses even more of a problem for the nearly 36 million American adults who lack basic literacy skills needed for employment [10] and for patients that possess little to no English reading proficiency.

Provider perception of open notes must also be considered as changes are implemented across healthcare systems. Primary care physicians report concern about the extra time required to construct and respond to note changes [2] and McCleary and colleagues [11] reported that oncologists viewed open notes in a positive light but differed from other specialties in how they felt the availability of notes would be perceived by patients. Oncologists appear to be more likely than other physicians to think that patients who utilize open notes would feel in more control of their care (82% vs. 63%), but less inclined to think that patient safety would improve (22% vs. 32%), and less likely to believe that patients would take better care of themselves (15% vs. 30%). Given the differences among subspecialties and the nature of their practice, this study suggests that open notes may not affect all providers in the same way.

On the other hand, sharing notes with patients is potentially beneficial for supporting patient autonomy, [12] by allowing patients to be more informed and involved in their care even if it does create new complexities within clinical encounters, especially for ethics consults. There have been longstanding concerns about how CEC notes should be documented, such as note length, what relevant details to include, and whether there should there be a clear ethics recommendation [13, 14]. Open notes requires consultants to also consider whether patients being able to quickly read the note will affect their perceptions of the case’s outcome, and if any changes to the note are ever warranted if, for example, a patient were to request to edit the note. An additional challenge relates to the documentation of cases in which the patient is not involved (e.g., a CEC focused on whether a patient should be offered a particular therapeutic option), such that they are learning about the CEC for the first time upon reading the note.

Some CEC services have sidestepped these questions by placing their notes behind internal firewalls, making them inaccessible to patients [15]. Notably, it is not clear whether the 21st Century Cures Act allows for such a carve-out for CEC notes, but a full exploration of this legal argument is beyond the scope of this manuscript. While this may be an option for some services, lack of availability of CEC notes violates the spirit of open notes and may risk losing the potential benefits for patient autonomy by not allowing patients to be as involved in ethical discussions about their care. In contrast, providing patients with access to CEC notes with open notes policies could serve as a steppingstone for healthcare systems to build new templates and revitalize existing ones that may be outdated, thereby serving as an opportunity to improve CECs. We concur with Mangino and Danis [15] who suggest that sharing CEC notes, with few exceptions, is likely beneficial for clinicians and patients alike.

## CEC documentation before open notes

There are numerous ways to document CECs, which usually follow from the process by which the CEC occurs. For example, Kaldjian and colleagues [13] proposed a method for CECs that mirrors a clinician’s approach to a traditional clinical case, and thus can serve as a model for notetaking as well. Rhodes and Alfandre [16] suggested a structured documentation method for consultation that is also closely aligned and structured according to traditional clinical reasoning, which can be adapted to inform the documentation of CECs. Orr and Shelton [17] offered another method of documentation intended to be helpful to all relevant stakeholders within a given ethics consultation, including patients, families, and the healthcare team. Table 1 presents a blended model that incorporates elements of all three approaches to documentation and generally aligns with current guidelines for healthcare ethics consultation provided by the American Society for Bioethics and Humanities [18].

While the order of documentation sections differs amongst the three methods described above, each includes similar general topics. All three gather relevant medical facts and history and use contextual features to formulate and answer ethics questions. In each, careful balancing occurs among competing ethical, legal, and individual principles, codes, and values. Evaluation, assessment, and discussion are used to consider relevant details to create a plan or recommendation. All processes leave open the possibility that there will be no clear conclusion, and more investigation, gathering of data, and deliberation may be necessary. These documentation processes serve as a framework upon which CEC open notes can potentially be improved.

## CEC documentation in the era of open notes

The movement to improve CEC processes and documentation practices has been a focus of attention for some time, especially the development of a national standardized credentialing program and more rigorous quality standards for ethics consultation [19]. To this end, many recommendations for CECs propose standardized, methodical ways to approach CEC and documentation with a general trend toward conformity. While standardization is useful, rigid adherence to documentation guidelines may not properly make sense of, or adequately address, the range of complexities involved in CEC cases in the era of open notes.

One potential method to strengthen the effectiveness of CEC notes and ensure sensitivity towards all readers is to incorporate aspects of narrative medicine into CEC documentation. Narrative medicine draws on the study and interpretation of stories to invigorate clinicians’ understanding of patient illness, filling it with more context and meaning. In the words of Rita Charon, a narrative medicine expert, “The capacity to recognize, absorb, metabolize, interpret, and be moved by stories of illness. Simply, it is medicine practiced by someone who knows what to do with stories” [20]. A strength of a narrative approach, with its compelling use of patient stories, is that patients who read their CEC notes may be more likely to understand and/or help the clinical team to “adjust the narrative” when appropriate. Indeed, CEC notes are stories – they summarize a narrative of an ethical problem that has arisen because of conflicting goals or values amongst patients, family members, and/or the healthcare team. Traditionally the CEC is thought of as a process for resolving conflict but viewing ethics consultation as a way to work together to construct a unified patient narrative may also be a useful lens to approach CECs and documentation.

There are many relevant stakeholders in any given CEC, with the primary concern typically directed at the patient involved; however, the narrative is often being told from the perspective of the CEC committee that is not involved in the patient’s direct care, and therefore, story. When reading a CEC note it must be considered then, “Who is telling the tale? From whose perspective are we hearing it? Whose story is it?” [21] Incorporating narrative prompts/questions into the documentation process could provide a way for CEC committee members to better decipher a patient’s goals, preferences, ideals, and beliefs, ensuring the patient’s story is not misrepresented or even worse, entirely left out. Indeed, narrative ethicist Martha Montello proposes the creation of an individualized “mattering map” [21] to characterize the most important people, relationships, places, and events in a patient’s life. The creation of such a map can be thought of as a conceptual exercise to focus attention to the morally relevant values of a patient and/or family, specifically where ruptures may have occurred due to illness.

Such a narrative-based, patient-centered approach could help address some of the concerns associated with CEC notes in the open notes era. Specifically, narrative approaches may foster patients’ ability to process the documentation and may even help ameliorate patient and clinician moral distress in the setting of CEC outcomes in which there is discord or disagreement. Open notes also creates an opportunity to consider the relationship between the CEC process and its documentation. Traditionally, the documentation occurs at the end of the CEC, and is not iterative. Yet, sharing notes with patients allows for a natural back-and-forth process, which provides patients an opportunity to seek clarification and/or changes. For instance, if a patient notices their perspectives have been misunderstood, or there are errors in documentation, they may have an opportunity to engage in the consultation process by helping correct the note. This iterative narrative process may also naturally contribute to conflict mediation by means of ensuring the “facts” of a given case are agreed upon. Case details inform the ethical analysis and potential recommendations given by a CEC service, and therefore have a higher potential to be erroneous without input from the patient and family. Careful reading and close listening are narrative concepts that can be incorporated into this process to improve accuracy, mediation, and the entire CEC. This process could meaningfully address errors present in the clinical notes from which much of the ethics narrative is taken. Indeed, a 2020 study by Bell and colleagues [22] found that 20% of patients who read one of their ambulatory notes found a mistake, 40% of which considered the mistake to be “serious.”

Another narrative approach would be to create a multi-voice narrative within a CEC note. Currently, the documentation process is centered around the perceived clinical perspective of the patient. Although the patient should be the primary concern, and primary voice within any given encounter, having the story of a CEC told from this singular perspective may not allow for the voices of all relevant stakeholders to be considered. The full expression of what contributes to conflict within a CEC may be better captured by recording different narrative viewpoints. In practice, a multi-voice narrative could appear in the form of collecting and recording the stories of multiple stakeholders in a single note, or even the creation of separate notes.

On the other hand, incorporating narrative features into CEC documentation can be time-consuming, subjective, and not necessarily action-guiding. Further, it may not be as valuable for clinicians who have no training or familiarity with the practice. Dedicated research is needed to examine how/whether implementation of a narrative framework improves CECs and the note-taking process. One simple method could be to compare patient satisfaction surveys following CECs that use a more traditional CEC framework to those that actively incorporate narrative elements. Regardless, if patients are to have access to CEC notes, as much effort as possible should be put into actively incorporating patient voices into the documentation, and to be sure narrative components can play a helpful role in this process. It is unlikely that a narrative approach would ever supplant the traditional models in Table 1, nor would this be desirable, but instead including aspects of narrative medicine into ethics notes could augment traditional CEC documentation methods rather than either method standing alone (see Table 2).

We have focused primarily on what is included, or what should be included, in CEC notes that are shared with patients. It is also worth considering when a note, or what aspects of a note, should *not* be shared with a patient. Mangino and Morris [15] propose two situations in which the sharing of a note with a patient would be unnecessary or harmful. First, if the CEC is related to an issue surrounding hospital/institutional policy, which has little to do with a specific patient’s care (e.g., a CEC broadly addressing research protocols or institutional scarcity arising from a distant clinical case), the note should not be shared. Second, if the consult addresses a topic about whose knowledge is anticipated to be disproportionately harmful to the patient, such as disclosure of unactionable or unwanted genetic data, sharing related notes is not recommended (in concordance with the principle of non-maleficence). These rare cases may entail double record keeping, in which some or all aspects of a note are not accessible to the patient, creating conflicting records of the CEC encounter. This process could potentially undermine the transparency and trust that open notes hopes to build, especially if patients learn about double, or restricted, ethics records. It might also create legal ramifications depending on how the creation and access of such records are interpreted under the Cures Act. Instead, it may be appropriate in some cases to not document aspects of a CEC encounter at all; indeed, there are aspects of all clinical encounters that regularly go undocumented. There is clearly subjectivity in these determinations, and discretion should be left to the ethics consultants and/or committee to determine whether they apply to a given case. These exceptions notwithstanding, steps should be taken to maintain open note access for CECs whenever possible as long as access would not cause undue harm to the patient (while recognizing that such exceptions run the risk of becoming an ethically treacherous slippery slope) [15].

## Conclusion

Open notes further complicates the practice of CEC documentation, but it also offers significant benefits of transparency, relational trust building, and supports patient autonomy. The 21st Century Cures Act provides for open notes because, overall, patients seem to benefit, and healthcare systems now have the ability to leverage this practice to also improve the CEC documentation process. Sparse research has focused on the documentation process of CECs [15], and specific data on how to best do this in the context of open notes are sorely needed. CECs are stories of ethical complexity and incorporating narrative medicine into notes could be one way to improve the CEC open notes process. The new regulatory requirements of the 21st Century Cures Act provide a valuable opportunity to involve patients more actively in the documentation and telling of their own story of ethical complexity. Healthcare systems should take advantage of this new era and use open notes as a way to improve CECs documentation and, ultimately, patient care.

**Table 1:** Clinical Ethics Consultation Documentation Elements [13, 16, 17]

Clinical Ethics Consultation Documentation Elements
*1) Gather Data* Relevant Clinical Facts -Demographic -Medical -Socioeconomic -Psychosocial Stakeholder Values -Religion -Beliefs -Preferences Relevant Local/Institutional Policies -Institutional -Legal
*2) Assess Whether the Problem is Ethical in Nature*
*3) Identify Relevant Ethical Principles, Duties, and/or Concepts* -Autonomy, beneficence, non-maleficence, and justice -Veracity -Duty to provide care -Fidelity -Confidentiality -Professional ethics
*4)Determine Whether Additional Data and/or More Discussions are Necessary*
*5) Provide an Overall Ethical Assessment* -Brief summary of clinical situation -Identification of related ethical issues
*6) Present Ethical Plan of Action Supported by and Balancing of*: -Principles -Rights -Consequences -Casuistry -Professional standard of practice -Conscientious practice -Institutional and legal policies
*7) Evaluate and Confirm Coherence of Conclusion*
*8) Provide Ethics Recommendation(s)*

**Table 2:** Narrative Elements of Clinical Ethics Consultation Documentation [21]

Narrative Elements
*Voice* -Who is telling the tale? -Whose perspective are we hearing? -Why is this story being told?
*Character* -Who is the center of the tale? -Whose story is it? -Are there missing voices/characters?
*Plot* -What has been disrupted in the story? -What has disrupted the integrity of the person and their story?
*Resolution* -Movement from dissonance to consonance -CECs do not end for patients at the conclusion of a consult -Find least damaging path forward -Restore integrity/wholeness of patient, family, and providers -Does the resolution fit in light of the participants’ stories?

## Data Availability

The datasets used and/or analyzed during the current study are available from the corresponding author on reasonable request.
